# Correction to: Python interfaces for the Smoldyn simulator

**DOI:** 10.1093/bioinformatics/btad558

**Published:** 2023-09-30

This is a correction to: Dilawar Singh, Steven S Andrews, Python interfaces for the Smoldyn simulator, *Bioinformatics*, Volume 38, Issue 1, January 2022, Pages 291–293, https://doi.org/10.1093/bioinformatics/btab530

In the originally published version of this manuscript, the top panel including software code was omitted from Figure 1.

**Figure 1.**
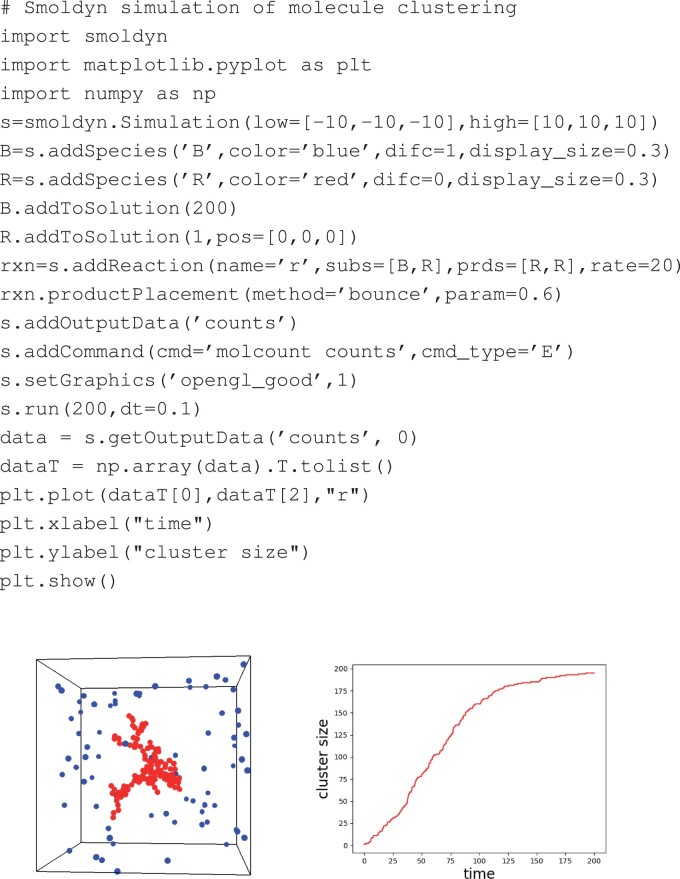
(Top) Complete Python code for a simple model of molecule clustering in which blue molecules (‘B’) diffuse freely, but then convert to immobile red molecules (‘R’) upon collision with a red molecule. (Left) A snapshot of a simulation from this model. (Right) The number of red molecules over time, from the same script (Color version of this figure is available at *Bioinformatics online*.)

This error has been corrected online.

